# Feasibility study of automated cardiac motion quantification to assess left ventricular function in type 2 diabetes

**DOI:** 10.1038/s41598-023-28262-3

**Published:** 2023-01-20

**Authors:** Yiming Li, Cungang Wu, Yuhong Li

**Affiliations:** Department of Medical Ultrasonics, First Affiliated Hospital With Jinzhou Medical University, No. 2, Section 5, Renmin Street, Guta District, Jinzhou, 121001 Liaoning Province China

**Keywords:** Cardiology, Endocrinology, Medical research

## Abstract

The global incidence of diabetes and related complications is gradually increasing, with cardiovascular complications being the leading cause of death in the diabetic population. The purpose of this study was to examine left ventricular function in individuals with type 2 diabetes mellitus (T2D) and conduct a feasibility analysis using automated cardiac motion quantification (aCMQ) approach. A total of 150 T2D patients with a history of diabetes mellitus dating back more than 10 years were chosen, and we treated 87 patients with T2D that had been present for less than 15 years as group I, 63 patients with T2D that had been present for more than 15 years as group II, and 50 healthy volunteers as the control group. From the three groups, clinical information, conventional ultrasonography parameters, and mitral annular plane systolic excursion (MAPSE) parameters were gathered. aCMQ technique was used to collect longitudinal strain and circumferential strain in the left ventricle. Tissue motion mitral annular displacement technique (TMAD) in aCMQ was used to collect parameters related to TMAD, and cardiac motion quantification (CMQ) was used to collect two-dimensional global longitudinal strain (2D-GLS) to compare the degree of difference between the aforementioned three groups. The differences between longitudinal strain groups in aCMQ were all statistically significant and gradually decreased with increasing disease duration. Most TMAD parameters were lower in groups I and II than in the control group, and TMAD parameters gradually decreased with increasing disease duration. The results of the LV global longitudinal strain and 2D-GLS using Bland–Altman analyses showed high agreement between and within groups, Pearson correlation analysis showed a significant positive correlation (r = 0.18, *P* < 0.05), and the AUC of ROC curves predicting the value of left ventricular function in patients with T2D was 0.723 and 0.628, respectively. With significant positive correlations between MAPSE, s', and the majority of the TAMD parameters (*P* < 0.05), TAMD, MAPSE, and s' demonstrated high inter- and intra-group agreement using Bland–Altman analyses, and the three had predictive value in assessing left ventricular function in T2D patients by ROC curve. Reduced longitudinal strain and reduced mitral annular displacement were seen in patients with different disease stages of T2D, so the application of aCMQ and TAMD was effective in detecting altered left ventricular function in patients with T2D. aCMQ had higher value in predicting left ventricular function in patients with T2D compared to CMQ for overall longitudinal strain, and the software performed the depiction automatically, reducing manual errors. MAPSE parameters and s ' can replace the TMAD technique for assessing mitral annular motion and was simpler to perform, saving operational time.

## Introduction

The global incidence of diabetes is gradually increasing, and the conditions of chronic hyperglycemia and insulin resistance can result in chronic lesions in various organs including the heart. Patients with diabetes usually experience various complications such as arterial atherosclerosis, diabetic foot, and coronary heart disease. Diabetic cardiomyopathy is one of the complications associated with diabetes and is a type of myocardial damage independent from the heart diseases induced by conditions such as coronary artery disease and hypertension^1^. The pathological manifestations of diabetic cardiomyopathy include cardiomegaly, left ventricular hypertrophy, and diffuse myocardial fibrosis^2^. The mechanism mediating myocardial cell damage in patients with diabetes is a complex composition of enhanced oxidative stress, endoplasmic reticulum stress, myocardial fibrosis, and activation of cardiac cell death pathways^3^. Altered myocardial energetics is a major risk factor for cardiac cell damage in patients with diabetes. The increased glucose and fatty acid levels lead to lipid deposition in the non-adipose tissues of the heart, resulting in lipotoxicity that exacerbates cellular damage^4–6^. Moreover, excessive glucose and fatty acids are used as substrates for the tricarboxylic acid cycle, causing a backlog of electrons in the respiratory chain that can leak and react with molecular oxygen to generate reactive oxygen species (ROS)^7,8^. Studies have shown that an increased level of ROS is essential to the pathology of various complications of diabetes^9^. During the subclinical period of diabetic cardiomyopathy, the manifestations in the heart are cardiac diastolic or systolic dysfunction, with the main triggers of the onset of diabetic cardiomyopathy being insulin resistance and the resulting hyperglycemia^10^. The common cardiac function evaluation modalities in patients with diabetes include three-dimensional (3D) echocardiography, electrocardiography (ECG), or transesophageal echocardiography, all of which have low sensitivity and can detect only markedly reduced cardiac function or altered cardiac structure in patients with diabetes who have often progressed to advanced stages of the disease. Early detection of altered cardiac function in patients with type 2 diabetes (T2D) is crucial as pharmacological intervention cannot restore impaired cellular function in the later stages of myocardial damage in patients with diabetes. Tools such as tissue Doppler imaging (TDI), strain stratification, and 2- or 3D speckle tracking were commonly used by previous researchers to study left ventricular (LV) function in patients with T2D. Although TDI has a higher sensitivity in assessing local myocardial motions despite the influences of the cardiac preload, afterload, and the heart itself, it is susceptible to angular limitations and machine gain^11^. Echocardiographic automated cardiac motion quantification (aCMQ) is a technique that can be used to analyze LV systolic function from a strain perspective based on a speckle-tracking technique that enables analysis of the longitudinal and circumferential strain at different LV myocardial segments. Tissue mitral annular displacement (TMAD) is used to assess the maximum displacement of the valve by measuring the absolute motion of the two insertion sites on the mitral annulus and the fixed apical location^12^. The first purpose of this research was to assess the left ventricular subclinical status of T2D patients of various durations using aCMQ and TMAD techniques. The second goal was to examine the viability of using aCMQ and CMQ to evaluate left ventricular function in T2D patients. The third objective was to investigate the viability of using MAPSE parameters, s', and TAMD parameters to evaluate left ventricular function in T2D patients.

## Materials and methods

### Study population and control subjects

150 patients with T2D who had been diagnosed in the Department of Endocrinology of the First Affiliated Hospital of Jinzhou Medical University between January 2021 and July 2022, met the diagnostic standards for type 2 diabetes set forth by the American Diabetes Association (ADA) in 2021^13^, and had a history of diabetes for more than 10 years were chosen. All of the patients had been diagnosed by an experienced endocrinologist. There were two groups based on the clinical history of diabetes mellitus: group I, which included 48 females and 39 men, had a mean age of (46.21 ± 5.82) years, ranged in age from 30 to 56, and had a disease duration of 10 to 15 years. In group II, there were 23 men and 40 women, aged 40 to 63, with a mean age of (52.22 ± 6.49) years and a disease duration of more than 15 years. Exclusion criteria for this study were cardiovascular diseases such as hypertension, coronary artery disease, heart failure, congenital heart disease, and heart valve disease; renal diseases such as primary glomerular disease, renal artery stenosis, and uremia; diagnosis of type 1 diabetes; poor-quality images; pregnancy; and malignancy treated using chemo- or radiotherapy. A total of 221 patients with T2D were recruited, and 71 patients with myocardial infarction (n = 8), heart failure (n = 5), hypertension (n = 51), malignancy (n = 1), and poor sonolucency (n = 6) were excluded.

Another 50 healthy volunteers were selected as controls from the same research period, and all were cleared from cardiac and renal diseases, hypertension as well as diabetes by ECG, echocardiography, and tests for blood pressure and biochemical indices. The control group consisted of 24 men and 26 women (average age, 47.46 ± 9.45 years), which was not statistically significantly different from the diabetic group (*P* > 0.05). A total of 57 healthy volunteers were recruited with seven excluded because of poor imaging quality.

Venous blood samples were collected in the morning from all subjects in a fasting state for testing biochemical indices. Age, body mass index (BMI), body surface area (BSA), heart rate, systolic blood pressure, diastolic blood pressure, gender composition, and smoking status were recorded.

Informed consent was obtained from all subjects. The personal information of all subjects was recorded and kept confidential, and the subjects received sufficient written or verbal instructions so that they could object to the use of their data for this study. All investigations were conducted following the principles of the Declaration of Helsinki.

### Echocardiography

A Philips EPIQ 7C color Doppler diagnostic ultrasound system (Philips, Best, The Netherlands) equipped with an S5-1 sector probe with a bandwidth of 1–5 MHz was used. After 30 min of rest, ECG leads were connected to each subject in all groups who were placed in the left lateral recumbent position, and all subjects were examined with the same diagnostic ultrasound system in the same clinic. All echocardiographic data acquisition and analyses were performed by an experienced sonographer. Before acquiring the images, optimizations were made by adjusting gain or depth based on the quality of the images. Conventional echocardiography was used to measure the interventricular septal thickness (IVST) and LV posterior wall thickness (LVPWT) in the parasternal long axis view of the LV in both groups. Measurements were performed per the guidelines of the American Society of Echocardiography. LVEF, LV end-diastolic volume (LVEDV), and LV end-systolic volume (LVESV) were measured in apical four-chamber views and two-chamber views using the biplane Simpson method. Pulsed Doppler was used to assess the early mitral orifice diastolic flow velocity (E), tissue Doppler was used to measure the peak mitral annular early diastolic motion (e') and the peak mitral systolic motion (s') in the lateral wall, and E/e' was determined. The peak longitudinal mitral annular displacement from end-diastole to end-systole was measured, and the two values were averaged, in an apical 4-chamber view using an M-mode ultrasound-adjusted sampling line placed at the root of the mitral annulus on the lateral wall and septal side of the left ventricle. The 2D dynamic images of apical four-chamber, three-chamber, and two-chamber views, as well as short-axis views at the LV mitral valve, papillary muscle, and apex levels from at least three cardiac cycles were acquired and stored on the diagnostic echocardiographic system. Images from each patient were acquired and stored at least three times so the optimal images could be selected for analysis.

All acquired images were imported into Qlab software for offline analysis with an aCMQ plug-in selected. Apical four-chamber, three-chamber, and two-chamber views were selected, and the regions of interest (ROI) were outlined automatically by the software. Further modifications could be made manually on the ROI generated automatically by the software until they were overlapping with the outlines of the endocardium and epicardium. When all analyses were finished, the “compute” button was clicked to calculate the longitudinal strain at different LV myocardial segments and generate bull’s-eye charts. In aCMQ, the short-axis views at the LV mitral valve, papillary muscle, and apex levels were analyzed one by one to obtain satisfactory ROI followed by calculating the circumferential strain and generating bull’s-eye charts using the same method described in the longitudinal strain analysis. The apical four-chamber and two-chamber views were loaded into the TMAD plug-in in aCMQ. In the apical four-chamber view, the tracking points were set at the septal site of the mitral annulus with left clicks, the lateral site of the mitral annulus, and the apex to obtain the displacement of the septal annulus toward the apex (AP4MV1), displacement of the lateral annulus toward the apex (AP4MV2), mean displacement of the mitral annulus in a four-chamber view toward the apex (AP4MV Midpt), and proportional displacement of the mitral annulus in a four-chamber view toward the apex (AP4MV Midpt%). In the apical two-chamber view, the three tracing points were placed at the inferior site of the mitral annulus, the anterior site of the mitral annulus, and the apex to yield displacement of the anterior annulus toward the apex (AP2MV1), displacement of the inferior annulus toward the apex (AP2MV2), mean displacement of the mitral annulus in a two-chamber view toward the apex (AP2MV Midpt), and proportional displacement of the mitral annulus in a two-chamber view toward the apex (AP2MV Midpt%). All measurements were repeated three times and averaged.

Apical four-chamber views, apical three-chamber views, and apical two-chamber views were imported into the CMQ, and the endocardial border was manually traced at end-diastole and manually adjusted to best match the endocardial position if necessary to derive a two-dimensional overall longitudinal strain.

### Repeatability test

In 30 patients randomly selected from the diabetic group, LVGLS, AP4MV1, s’, MAPSE-mean, and 2D-GLS were analyzed by two independent physicians who were skilled in the use of Qlab software. The two physicians were blinded to each other’s data, and the data collection process was repeated by one of the physicians 1 week later. The inter-observer and intra-observer variability were examined with the Bland–Altman tests.

### Statistical analysis

SPSS 23.0 statistical software was applied for statistical analysis, and the chi-square test was used for the comparison of count data. All measurement data were tested for normality, and those conforming to normal distribution were expressed as mean ± standard deviation, and the F test was used for intergroup comparison between groups, and the LSD-t test was used for two-way comparison between groups. Data that did not conform to a normal distribution are presented as medians and interquartile ranges and were compared using the Kruskal–Wallis rank sum test for differences between groups. Pearson correlation analysis was used to analyze the correlation of TMAD parameters with MAPSE parameters and s'. The area under the ROC curve (AUC) was computed after the receiver operating curve (ROC) of the aCMQ parameters, TMAD parameters, MAPSE parameters, s', and 2D-GLS in predicting left ventricular systolic function of T2D was drawn. The prediction was considered unreliable with minimal diagnostic value when the AUC was < 0.5, some diagnostic value when the AUC was > 0.5, and a high diagnostic value when AUC was > 0.7. A difference with *P* < 0.05 was considered statistically significant.

### Ethical approval

The study was approved by the Ethics Committee of the First Affiliated Hospital of Jizhou Medical University (Jinzhou, China) (202252).

### Informed consent

The following information was obtained from all participants.

## Results

### Comparison of general clinical characteristics

BMI, BSA, heart rate, systolic blood pressure, diastolic blood pressure, and gender composition were not statistically significant between the groups (*P* > 0.05). The number of smokers, fasting state glucose, HbA_1c_, triglycerides, and total cholesterol were higher in group I than in the control group, and the high-density lipoprotein level (HDL) was lower than in the control group (*P* < 0.05). Age, glucose, glycosylated hemoglobin, low-density lipoprotein level (LDL), triglycerides, and total cholesterol were higher in group II than in the control group, and HDL was lower than in the control group (*P* < 0.05). Age and LDL were higher in group II than in group I, and the number of smokers was lower than in group I (*P* < 0.05) (Table 1).

**Table 1:** Clinical and laboratory parameters. BMI, body mass index; BSA, body surface area; HbA_1c_, glycated hemoglobin; HDL, high-density lipoprotein; LDL, low-density lipoprotein.

Parameter	Group I	Group II	Control	*P* value
Age (years)	46.21 ± 5.82	52.22 ± 6.49*^,#^	47.46 ± 9.45	0.000
BMI (kg/m^2^)	25.40 ± 3.06	24.59 ± 3.91	24.33 ± 3.49	0.163
BSA (m^2^)	1.74 ± 0.10	1.72 ± 0.09	1.74 ± 0.14	0.544
Heart rate (beats/min)	74.33 ± 7.52	73.84 ± 5.46	74.98 ± 11.54	0.764
Systolic pressure (mm/Hg)	119.36 ± 6.01	118.89 ± 5.54	118.88 ± 7.52	0.871
Diastolic pressure (mm/Hg)	73.03 ± 3.29	74.24 ± 3.31	72.70 ± 4.46	0.051
Gender (male, %)	39 (44.83%)	23 (36.51%)	24 (48%)	0.425
Smoking	51 (58.62%)*	27 (42.86) ^#^	16 (32%)	0.008
Fasting plasma glucose (mmol/l)	10.75 ± 2.48*	10.91 ± 1.75*	4.70 ± 0.56	0.000
HbA_1c_ (%)	8.71 ± 6.29*	8.16 ± 1.46*	4.44 ± 0.31	0.000
HDL (mg/dL)	1.141 (1.0,1.3)*	1.040 (1.0,1.1)*^,#^	1.185 (1.0,1.4)	0.000
LDL (mg/dL)	3.12 ± 0.63	3.39 ± 0.42*^,#^	2.92 ± 0.90	0.001
Triglycerides (mmol/L)	1.98 ± 1.10*	2.08 ± 1.10*	1.39 ± 0.53	0.001
Total cholesterol (mmol/L)	5.01 ± 0.84*	4.99 ± 0.71*	4.43 ± 1.08	0.000

Data are presented as mean ± standard deviation, median (interquartile range), or number (%).^*^Compared with control group, *P* < 0.05. ^#^Compared with group I, *P* < 0.05.

### Comparison of conventional echocardiographic parameters

The data were similar between the groups of LVESV, LVEDV, IVST, LVPWT, and E (*P* > 0.05). e' was lower in group II than control and group I, and E/e' was higher than control and group I (*P* < 0.05). LVEF was higher in group II than in control (*P* < 0.05) (Table 2).

**Table 2:** Conventional echocardiographic parameters. Data are presented as mean ± standard deviation. LVESV, left ventricular end-systolic volume; LVEDV, left ventricular end-diastolic volume; LVEF, left ventricular ejection fraction; IVST, interventricular septal thickness; LVPWT, left ventricular posterior wall thickness. *Compared with control group, *P* < 0.05. ^#^Compared with group I, *P* < 0.05.

Parameter	Group I	Group II	Control	*P* value
LVESV (ml)	32.74 ± 3.75	33.57 ± 4.40	32.08 ± 7.26	0.290
LVEDV (ml)	92.59 ± 7.21	93.31 ± 7.00	89.73 ± 14.73	0.121
LVEF (%)	63.52 ± 2.76	63.00 ± 2.45*	64.57 ± 4.29	0.029
IVST (mm)	0.90 ± 0.05	0.91 ± 0.05	0.89 ± 0.06	0.234
LVPWT (mm)	1.00 ± 0.05	0.99 ± 0.05	0.99 ± 0.03	0.444
E (cm/s)	79.10 ± 16.48	76.93 ± 10.59	80.59 ± 12.41	0.366
e′ (cm/s)	11.57 ± 1.02	10.18 ± 0.93*^,#^	11.82 ± 1.54	0.000
E/e′	6.88 ± 1.44	7.63 ± 1.33*^,#^	6.94 ± 1.41	0.004

### Comparison of strain parameters

There was no statistically significant difference between the groups in the circumferential strain evaluated at the basal left ventricular level (SAXBCS) and the global left ventricular circumferential strain (LVGCS) (*P* > 0.05). LVGLS, longitudinal strain of the left ventricle in apical two-chamber view (LVAP2LS), longitudinal strain of the left ventricle in apical three-chamber view (LVAP3LS), longitudinal strain of the left ventricle in apical four-chamber view (LVAP4LS), and 2D-GLS in group I were significantly lower than those in the control group (*P* < 0.05). LVGLS, LVAP2LS, LVAP3LS, LVAP4LS, and 2D-GLS in group II were significantly lower than those in control and group I (*P* < 0.05). Circumferential strain measured at the basal left ventricular level (SAXMCS) and circumferential strain measured at the apical left ventricular level (SAXACS) were lower in group II than in the control group (*P* < 0.05). The differences between groups for the remaining parameters were not statistically significant (Table 3).

**Table 3:** Strain analysis. Data are presented as mean ± standard deviation. LVGLS, left ventricular global longitudinal strain; LVAP2LS, longitudinal strain of the left ventricle in apical two-chamber view; LVAP3LS, longitudinal strain of the left ventricle in apical three-chamber view; LVAP4LS, longitudinal strain of the left ventricle in apical four-chamber view; SAXBCS, circumferential strain measured at the basal left ventricular level; SAXMCS, circumferential strain measured at the mid left ventricular level; SAXACS, circumferential strain measured at the apical left ventricular level; LVGCS, left ventricular global circumferential strain; 2D-GLS, two dimensional global longitudinal strain. *Compared with control group, *P* < 0.05. ^#^Compared with group I, *P* < 0.05.

Parameter	Group I	Group II	Control	*P* value
LVGLS (%)	− 13.92 ± 1.45*	13.39 ± 1.28*^,#^	− 16.22 ± 1.10	0.000
LVAP2LS (%)	− 13.98 ± 1.21*	13.19 ± 1.53*^,#^	− 15.58 ± 1.77	0.000
LVAP3LS (%)	− 14.62 ± 1.58*	12.96 ± 1.87*^,#^	− 16.27 ± 1.76	0.000
LVAP4LS (%)	− 15.79 ± 1.51*	14.42 ± 0.94*^,#^	− 17.01 ± 1.70	0.000
SAXBCS (%)	− 23.09 ± 2.69	22.33 ± 2.01	− 23.52 ± 3.70	0.070
SAXMCS (%)	− 25.27 ± 3.30	24.12 ± 2.83*	− 25.92 ± 5.81	0.047
SAXACS (%)	− 26.69 ± 3.55	25.43 ± 2.26*	− 28.29 ± 7.68	0.006
LVGCS (%)	− 25.27 ± 2.94	24.64 ± 2.53	− 25.56 ± 5.47	0.369
2D-GLS (%)	15.56 ± 1.07*	15.05 ± 1.19*^,#^	16.04 ± 0.94	0.000

### Comparison of TMAD parameters, MAPSE parameters and s'

AP4MV1, AP4MV2, AP4MV Midpt, AP4MV Midpt%, AP2MV2, AP2MV Midpt, AP2MV Midpt%, s', MAPSE-SEPTAL, MAPSE-LATERAL, and MAPSE-mean were significantly lower in group I than in the control group (*P* < 0.05). AP4MV1, AP4MV Midpt, AP2MV1, AP2MV2, AP2MV Midpt, AP2MV Midpt%, s', MAPSE-SEPTAL, MAPSE-LATERAL, and MAPSE-mean were significantly lower in group II than in group I and the control group (*P* < 0.05). Both AP4MV2 and AP4MV Midpt% were lower in group II than in the control group (*P* < 0.05) (Table 4).

**Table 4:** MAPSE, s’ and TMAD parameters. Data are presented as mean ± standard deviation. TMAD, tissue mitral annular displacement; AP4MV1, displacement of the septal annulus toward the apex; AP4MV2, displacement of the lateral annulus toward the apex; AP4MV Midpt, displacement of a midpoint in a four-chamber view toward the apex; AP4MV Midpt%, proportional displacement of a midpoint in a four-chamber view toward the apex (AP4MV Midpt/left ventricular end-diastolic internal diameter); AP2MV1, displacement of the anterior annulus toward the apex; AP2MV2, displacement of the inferior annulus toward the apex; AP2MV Midpt, displacement of a midpoint in a two-chamber view toward the apex; AP2MV Midpt%, proportional displacement of a midpoint in a two-chamber view toward the apex (AP2MV Midpt/left ventricular end-diastolic internal diameter). MAPSE, Mitral Annular Plane Systolic Excursion. *Compared with control group, *P* < 0.05. ^#^Compared with group I, *P* < 0.05.

Parameter	Group I	Group II	Control	*P* value
AP4MV1 (mm)	11.36 ± 1.55*	10.71 ± 1.41*^,#^	12.34 ± 0.94	0.000
AP4MV2 (mm)	12.14 ± 2.24*	11.57 ± 1.48*	13.66 ± 1.48	0.000
AP4MV Midpt (mm)	11.58 ± 1.37*	10.83 ± 1.29*^,#^	12.93 ± 1.56	0.000
AP4MV Midpt%	13.33 ± 1.77*	13.21 ± 1.43*	15.13 ± 1.17	0.000
AP2MV1 (mm)	11.91 ± 1.38	11.27 ± 1.04*^,#^	12.48 ± 2.58	0.001
AP2MV2 (mm)	11.83 ± 1.36*	11.13 ± 1.39*^,#^	12.87 ± 2.74	0.000
AP2MV Midpt (mm)	11.54 ± 1.23*	10.97 ± 1.37*^,#^	12.49 ± 2.39	0.000
AP2MV Midpt%	13.53 ± 1.85*	12.80 ± 1.55*^,#^	15.41 ± 3.07	0.000
s’(cm/s)	6.86 ± 0.51*	6.60 ± 0.43*^,#^	7.25 ± 0.82	0.000
MAPSE-SEPTAL (mm)	14.99 ± 1.07*	13.37 ± 1.79*^,#^	15.32 ± 0.72	0.000
MAPSE-LATERAL (mm)	15.33 ± 0.82*	14.55 ± 1.09*^,#^	15.82 ± 0.67	0.000
MAPSE-mean (mm)	15.19 ± 0.69*	13.98 ± 1.10*^,#^	15.60 ± 0.45	0.000

### Repeatability test

**Figure 1:**
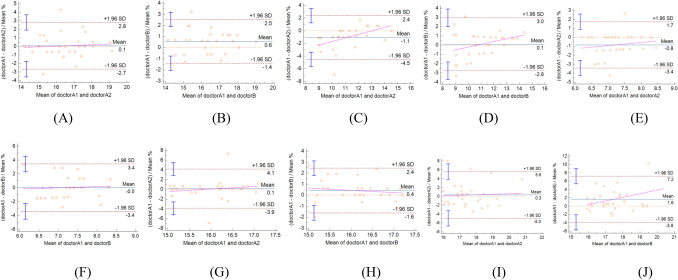
Bland–Altman analyses. Bland–Altman analysis of LVGLS showed that there was a high consistency in intraobserver (**A**). Bland–Altman analysis of LVGLS showed that there was a high consistency in interobservers (**B**). Bland–Altman analysis of AP4MV1 showed that there was a high consistency in intraobserver (**C**). Bland–Altman analysis of AP4MV1 showed that there was a high consistency in interobservers (**D**). Bland–Altman analysis of s’ showed that there was a high consistency in intraobserver (**E**). Bland–Altman analysis of s’ showed that there was a high consistency in interobservers (**F**). Bland–Altman analysis of MAPSE-mean showed that there was a high consistency in intraobserver (**G**). Bland–Altman analysis of MAPSE-mean showed that there was a high consistency in interobservers (**H**). Bland–Altman analysis of 2D-GLS showed that there was a high consistency in intraobserver (**I**). Bland–Altman analysis of 2D-GLS showed that there was a high consistency in interobservers (**J**).

The intra- and inter-observer agreement was high for LVGLS, AP4MV1, s', MAPSE-mean, and 2D-GLS (Fig. 1).

### Pearson correlation analysis

**Figure 2:**
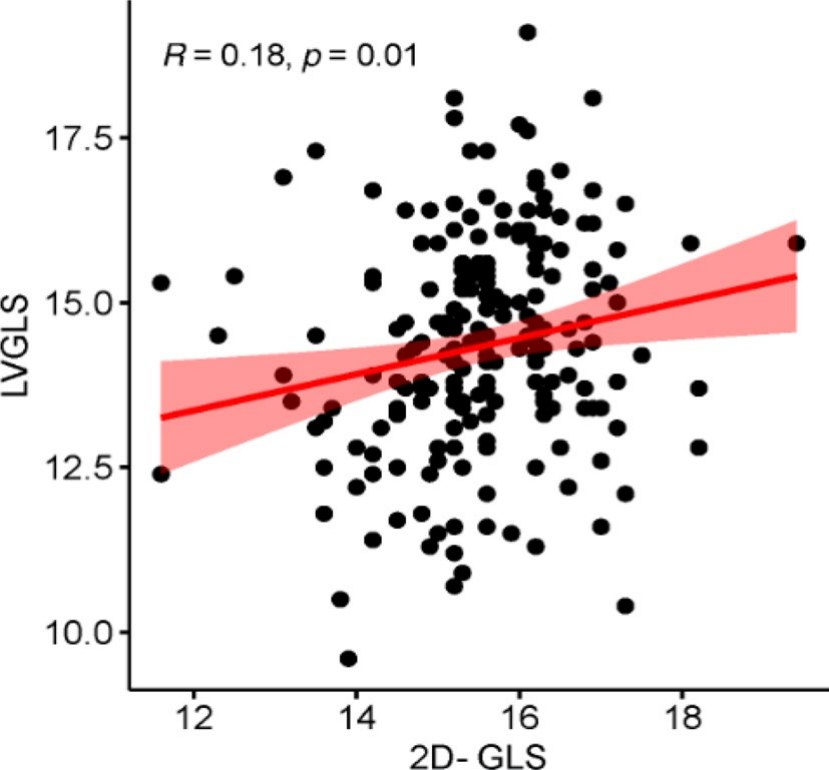
Correlation analysis of LVGLS with 2D-GLS. LVGLS, left ventricular global longitudinal strain; 2D-GLS, two dimensional global longitudinal strain.

MAPSE-SEPTAL was positively correlated with AP4MV Midpt, AP4MV Midpt%, AP2MV2, AP2MV Midpt (r = 0.175, 0.249, 0.173, 0.215, all *P* < 0.05). MAPSE-LATERAL and AP4MV1, AP4MV2, AP4MV Midpt, AP4MV Midpt%, AP2MV2, AP2MV Midpt, AP2MV Midpt% were positively correlated with each other (r = 0.268, 0.260, 0.280, 0.153, 0.164, 0.197, 0.261, all *P* < 0.05). MAPSE-mean was correlated with AP4MV1, AP4MV2, AP4MV Midpt, AP4MV Midpt%, AP2MV2, AP2MV Midpt, and AP2MV Midpt% were positively correlated with each other (r = 0.233, 0.228, 0.266, 0.258, 0.207, 0.256, 0.218, all *P* < 0.05). s' and AP4MV1, AP4MV2, AP4MV Midpt, AP4MV Midpt%, AP2MV1, AP2MV2, AP2MV Midpt% were positively correlated with each other (r = 0.151, 0.185, 0.255, 0.277, 0.231, 0.168, 0.208, all *P* < 0.05). There was no correlation between MAPSE-LATERAL and MAPSE-mean and AP2MV1 (r = 0.130 and 0.131, both *P* > 0.05). There was no correlation between s' and AP2MV Midpt (r = 0.111, *P* > 0.05). MAPSE-SEPTAL was not correlated with AP4MV1, AP4MV2, AP2MV1, AP2MV Midpt% (r = 0.136, 0.137, 0.090, 0.121, all *P* > 0.05) (Table 5). LVGLS was positively correlated with 2D GLS (r = 0.18, *P* < 0.05) (Fig. 2).

**Table 5:** Correlation analysis of TMAD parameters with MAPSE parameters and s’. TMAD, tissue mitral annular displacement; AP4MV1, displacement of the septal annulus toward the apex; AP4MV2, displacement of the lateral annulus toward the apex; AP4MV Midpt, displacement of a midpoint in a four-chamber view toward the apex; AP4MV Midpt%, proportional displacement of a midpoint in a four-chamber view toward the apex (AP4MV Midpt/left ventricular end-diastolic internal diameter); AP2MV1, displacement of the anterior annulus toward the apex; AP2MV2, displacement of the inferior annulus toward the apex; AP2MV Midpt, displacement of a midpoint in a two-chamber view toward the apex; AP2MV Midpt%, proportional displacement of a midpoint in a two-chamber view toward the apex (AP2MV Midpt/left ventricular end-diastolic internal diameter). MAPSE, Mitral Annular Plane Systolic Excursion.

	MAPSE-SEPTAL		MAPSE-LATERAL		MAPSE-mean		s’	
	R	*P* value	r	*P* value	r	*P* value	r	*P* value
AP4MV1	0.136	0.054	0.268	0.000	0.233	0.001	0.151	0.032
AP4MV2	0.137	0.053	0.260	0.000	0.228	0.001	0.185	0.009
AP4MV Midpt	0.175	0.013	0.280	0.000	0.266	0.000	0.255	0.000
AP4MV Midpt%	0.249	0.000	0.153	0.031	0.258	0.000	0.277	0.000
AP2MV1	0.090	0.207	0.130	0.066	0.131	0.065	0.231	0.001
AP2MV2	0.173	0.014	0.164	0.020	0.207	0.003	0.168	0.018
AP2MV Midpt	0.215	0.002	0.197	0.005	0.256	0.000	0.111	0.116
AP2MV Midpt%	0.121	0.087	0.261	0.000	0.218	0.002	0.208	0.003

### ROC curve analysis

The AUC of LVGLS, LVAP2LS, LVAP3LS, LVAP4LS, and 2D-GLS for evaluating left ventricular systolic function in T2D were 0.723, 0.676, 0.662, 0.689, and 0.680, respectively (Table 6). AP4MV1, AP4MV2, AP4 MV Midpt, AP4MV Midpt%, AP2MV1, AP2MV2, AP2MV Midpt, AP2MV Midpt%, s', MAPSE-SEPTAL, MAPSE-LATERAL, MAPSE-mean assessment the AUC of T2D LV systolic function were 0.827, 0.754, 0.787, 0.714, 0.691, 0.720, 0.704, 0.683, 0.679, 0.693, 0.743, and 0.786, respectively (Table 7).

**Table 6:** ROC curves of aCMQ parameters and 2D-GLS predicting LV systolic dysfunction in patients with T2D.

Parameter	AUC	Sensitivity	Specificity	Cut-off
LVGLS	0.723 (0.625~0.807)	0.840	0.615	− 15.300%
LVAP2LS	0.676 (0.576~0.765)	0.920	0.500	− 13.500%
LVAP3LS	0.662 (0.561~0.752)	0.980	0.423	− 13.100%
LVAP4LS	0.689 (0.589~0.777)	0.900	0.500	− 15.200%
2D-GLS	0.680 (0.602~0.759)	0.640	0.693	− 15.7%

**Table 7:** ROC curves of TMAD parameters, MAPSE parameters and s’ predicting LV systolic dysfunction in patients with T2D.

Parameter	AUC	Sensitivity	Specificity	Cut-off
AP4MV1	0.827 (0.739~0.894)	1.000	0.673	10.500 mm
AP4MV2	0.754 (0.658~0.834)	0.880	0.654	12.200 mm
AP4MV Midpt	0.787 (0.695~0.862)	0.800	0.673	11.500 mm
AP4MV Midpt%	0.714 (0.616~0.800)	0.980	0.481	13.000%
AP2MV1	0.691 (0.592~0.779)	0.760	0.558	10.800 mm
AP2MV2	0.720 (0.623~0.805)	0.880	0.538	10.800 mm
AP2MV Midpt	0.704 (0.605~0.790)	0.880	0.462	10.600 mm
AP2MV Midpt%	0.683 (0.584~0.772)	0.820	0.519	13.200%
s’	0.679 (0.585 ~ 0.774)	0.460	0.867	7.200 cm/s
MAPSE-SEPTAL	0.693 (0.620 ~ 0.765)	0.940	0.447	14.200 mm
MAPSE-LATERAL	0.743 (0.667 ~ 0.819)	0.580	0.807	15.600 mm
MAPSE-mean	0.786 (0.724 ~ 0.848)	0.880	0.867	15.000 mm

### Image analysis using aCMQ

**Figure 3:**
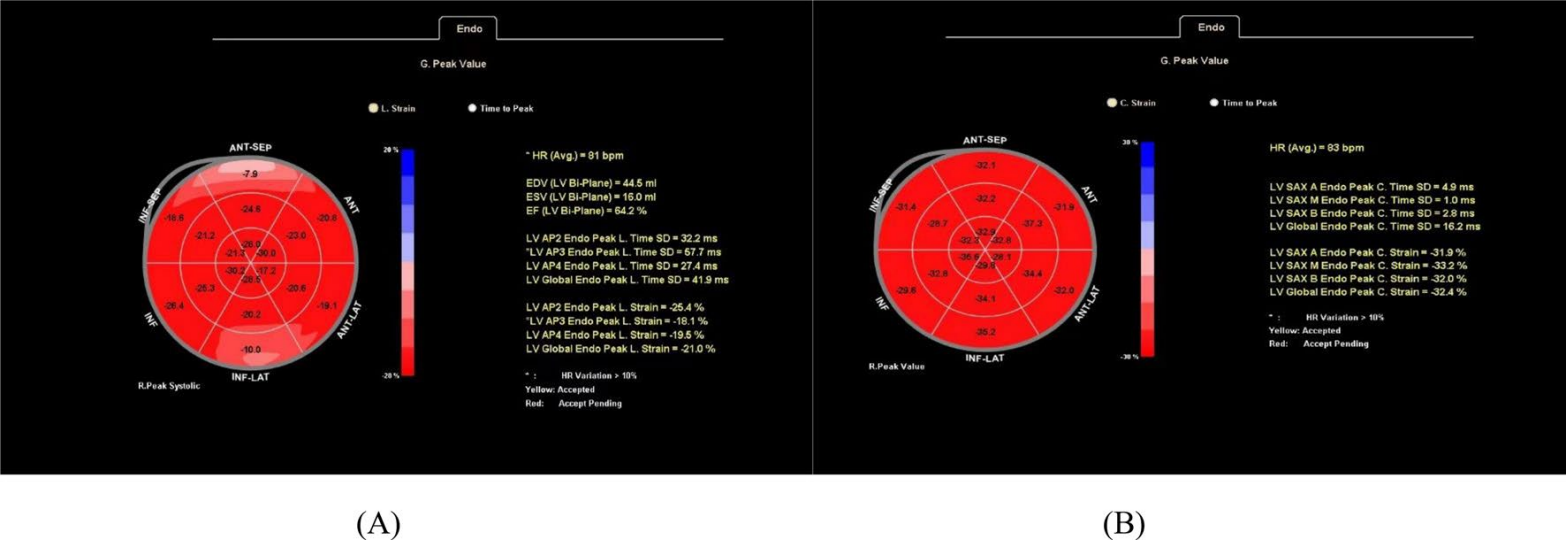
Bovine eye diagram of healthy control group. Bovine eye diagram of left ventricular longitudinal strain (**A**). Bovine eye diagram of left ventricular circumferential strain (**B**).

The bovine eye map of circumferential and longitudinal strains in the normal control group is shown in Fig. 3. The LVGLS and LVGCS were -21.0% and -32.4%, respectively, without a decrease in left ventricular systolic function, and the color distribution of the bovine eye map was consistent.

**Figure 4:**
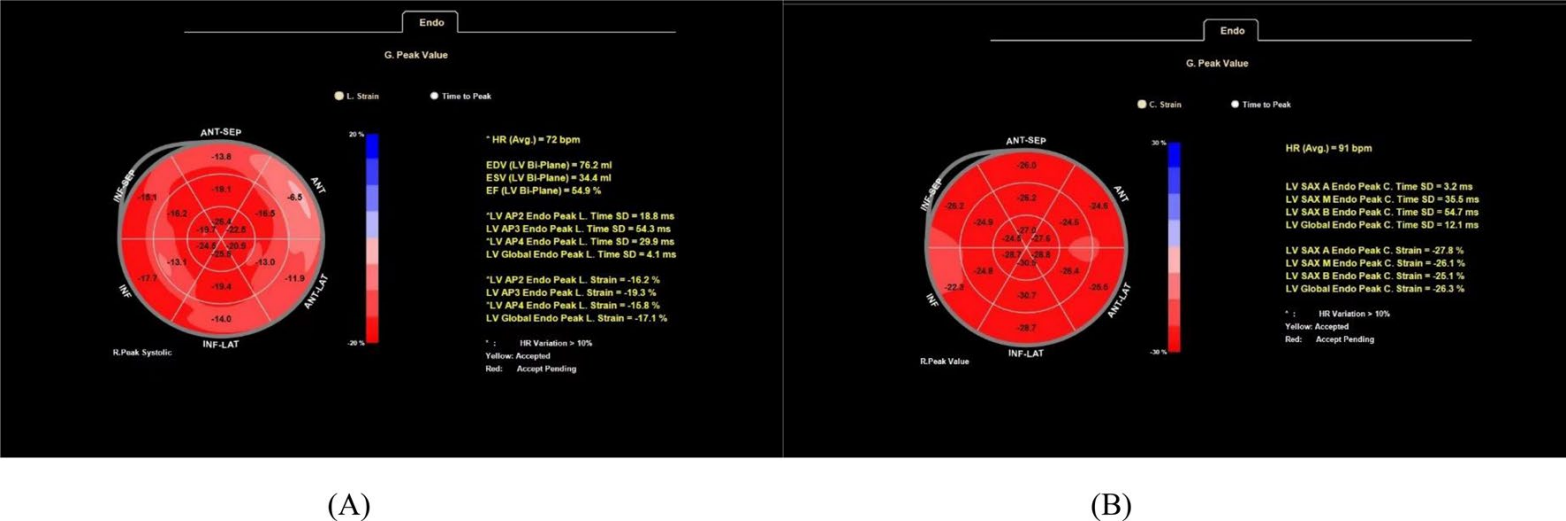
Bovine eye diagram of diabetic group. Bovine eye diagram of left ventricular longitudinal strain (**A**). Bovine eye diagram of left ventricular circumferential strain (**B**).

A patient with diabetes is depicted in the bovine eye map of annular strain and longitudinal strain in Fig. 4. The left ventricular systolic function was diminished, as seen by the uneven color distribution of the bovine eye map, which had LVGLS of − 17.1% and LVGCS of − 26.3%.

## Discussion

The global incidence of diabetes and associated complications is gradually increasing, with cardiovascular complications being the leading cause of death in the diabetic population^14^. Even after excluding myocardial damage caused by other factors such as hypertension and ischemic heart disease, patients with diabetes are more likely to develop cardiovascular disease than healthy people. The effects of diabetes on the heart are mostly characterized by heart failure in the later stages, a type of cardiomyopathy known as diabetic cardiomyopathy^15–17^. The incidence of heart failure is higher in a diabetic than in a nondiabetic population with a worse prognosis^18^. This is because the energy supply for hearts in patients with heart failure is more dependent on glucose oxidation, and insulin resistance in the diabetic population limits the energy supply, which further exacerbates heart failure. Meanwhile, heart failure is conducive to systemic insulin resistance, which further contributes to the poor prognosis in the diabetic population^19^. Therefore, early detection of myocardial damage in patients with diabetes is crucial. However, in the subclinical period, diagnosing diabetic cardiomyopathy with routine ultrasound is challenging because the cardiac function of patients with diabetes usually remains within the normal range with no specific ultrasound presentation. Previous studies have shown that the first manifestation of diabetic cardiomyopathy was diastolic dysfunction, followed by systolic dysfunction, and finally heart failure. However, Minciună et al.^20^ used the speckle-tracking technique to assess patients with diabetes in their subclinical status and confirmed the presence of LV systolic dysfunction in the subclinical period independent of the existence of LV diastolic dysfunction. The aCMQ technique was developed based on the speckle-tracking technique, in which the system automatically outlines the endocardium of the LV, calculates the peak strain, and generates bull’s-eye charts throughout the different phases of systole in the LV. It can accurately reflect LV function regardless of the direction of ventricular wall motion or heart motion. TMAD is another technique based on the speckle-tracking technique, which allows rapid tracking of the designated sites on the septal, lateral, inferior, and anterior mitral annulus without being influenced by endocardium or image clarity. It allows the evaluation of LV function as long as the mitral and apical locations are clearly displayed on the ultrasound images. TMAD has been demonstrated in several studies to be convenient and sensitive for evaluating LV function^12,21–23^.

In this study, LVEDV, LVESV, LVPWT, and IVST measured by conventional echocardiography remained within normal values and did not decrease significantly, and only LVEF was lower in group II patients compared with controls, which may be due to the subclinical state of the heart in diabetic patients. Previous studies have shown that in patients with normal LVEF, fibrotic myogenic degeneration of the cardiomyocytes may be a result of the increased oxidative stress, to which LVEF is insusceptible^24–26^. Patients in group II had lower e' and higher E/e' than controls and group I, reflecting that patients with T2D showed signs of LV diastolic dysfunction and that, as the disease progressively worsened, it would further cause LV diastolic dysfunction if LV compliance continued to decrease. Fang et al.^27^ found a higher incidence of LV diastolic and systolic dysfunction, manifested as abnormal velocity of LV myocardial contraction and relaxation, in patients with diabetes without myocardial ischemia or LV wall hypertrophy. With increasing disease duration, LVGLS, LVAP2LS, LVAP3LS, LVAP4LS, and 2D GLS strain values gradually declined. Longitudinal strain was lower in Group I patients than in the control group, and in Group II patients than in both Group I and the control group. Statistics showed that the differences were significant (*P* < 0.05). The differences between SAXBCS and LVGCS groups were not statistically significant (*P* > 0.05), and only SAXMCS and SAXACS were lower in group II compared with the control group. This indicated that longitudinal strain was more susceptible to change than circumferential strain in the LV of patients with diabetes. It is possible that the LV endocardium, the myocardial fibers of which are predominantly distributed longitudinally, is the first to be damaged by hyperglycemia or free fatty acids, which endows the longitudinal strain with higher statistical significance than circumferential strain^28^. This result is consistent with the reported findings of many previous studies. For example, a study on cardiac involvement in children with diabetes conducted by Motamedi et al.^29^ showed lower longitudinal strain in all segments of the heart. Sonaglioni et al.^30^ suggested that the overall longitudinal strain in the LV of patients with gestational diabetes was significantly lower than that in healthy pregnant women. The statistically significant differences between the AP4MV1, AP4MV Midpt, AP2MV2, AP2MV Midpt, AP2MV Midpt%s', MAPSE-SEPTAL, MAPSE-LATERAL, and MAPSE-mean groups gradually decreased with increasing disease duration. AP4MV2 and AP4MV Midpt% were lower in groups I and II compared with the control group, and AP2MV1 was lower in group II compared with the control group and group I. This could be because impaired cardiomyocyte viability or mitochondrial function in the patients with diabetes increased apoptosis and oxidative stress, which lead to reduced motion of the mitral annulus toward the apex during LV systole compared to that in healthy controls. The results of this study are similar to those reported by Iwakura et al.^31^.

We found that most TMAD parameters were significantly and positively correlated with MAPSE parameters and s', and all three Bland–Altman results showed high agreement, and all three had predictive value in assessing left ventricular function in patients with T2D, further suggested that MAPSE parameters and s' can be used as a proxy for TMAD parameters, but s' had a lower value in predicting left ventricular function compared to TMAD and MAPSE parameters. Using MAPSE parameters to analyze patients with Duchenne muscular dystrophy, Panovsk et al.^32^ discovered reduced left ventricular systolic function despite normal LVEF, demonstrating the viability of MAPSE in assessing left ventricular systolic function and correlating with the findings of the current study. According to our findings, there is a substantial connection between 2D GLS and LVGLS and excellent inter- and intragroup agreement between the two methodologies when measuring overall longitudinal LV strain in patients with T2D using automatic and manual methods. Compared to LVGLS measured mechanically, 2D GLS performed manually was less useful in predicting LV function in individuals with T2D. A comprehensive analysis of the feasibility of the aCMQ technique to assess left ventricular function in patients with T2D was better.

We believe that the findings of reduced LV longitudinal strain and mitral annular displacement in patients with T2D by aCMQ and TMAD suggested early myocardial damage in patients with diabetes, and clinicians should be informed to commence early intervention to ameliorate and stop the progression of diabetic cardiomyopathy. Many researchers have noted that exercise could help prevent cardiomyocyte apoptosis, myocardial fibrosis, and oxidative stress, which could moderately delay the development of diabetic myocardial damage^33–35^.

There are certain limitations to this study. First, the application of the aCMQ technique requires a clear depiction of the endocardium. However, in practice, the endocardium can appear unclear because of unavoidable factors such as smoking. In these cases, the endocardium needs to be outlined manually, which may generate operator error and deviations from the true values. Second, mitral valve degeneration or calcification in some patients may have influenced their mitral annular displacement. Finally, because of limited research duration and objective constraints, the patients with T2D selected for this study were regional and few.

## Conclusion

It is highly feasible for aCMQ and TMAD to evaluate the left ventricular systolic function in patients with T2D and correlate it with the duration of the patient's disease. This can serve as a foundation for the early clinical detection of myocardial damage changes in patients with T2D and for the prompt application of appropriate measures to prevent further development of myocardial injury.

## Data Availability

The datasets generated and/or analysed during the current study are not publicly available due to privacy or ethical restrictions but are available from the corresponding author on reasonable request.
